# Visual post-occupancy evaluation of a restorative garden using virtual reality photography: Restoration, emotions, and behavior in older and younger people

**DOI:** 10.3389/fpsyg.2022.927688

**Published:** 2022-08-30

**Authors:** Marco Boffi, Linda Grazia Pola, Elisabetta Fermani, Giulio Senes, Paolo Inghilleri, Barbara Ester Adele Piga, Gabriele Stancato, Natalia Fumagalli

**Affiliations:** ^1^Department of Cultural Heritage and Environment, University of Milan, Milan, Italy; ^2^Department of Agricultural and Environmental Sciences, University of Milan, Milan, Italy; ^3^Department of Architecture and Urban Studies, Politecnico di Milano, Milan, Italy

**Keywords:** healing garden, older society, attention restoration, emotional appraisal, behavioral effect, landscape design, virtual reality, post-occupancy evaluation

## Abstract

Natural environments have a restorative effect from mental/attentional fatigue, prevent stress, and help to revitalize psychological and physical resources. These benefits are crucial for promoting active aging, which is particularly relevant given the phenomenon of population aging in recent decades. To be considered restorative, green spaces have to meet specific requirements in ecological and psychological terms that can be assessed through Post-Occupancy Evaluation (POE), a multimethod approach commonly used by environmental psychologists and landscape architects after construction to evaluate the design outcomes from the users’ perspective. Generally, POEs consist of surveys and/or interviews accompanied by more or less structured observations of onsite users’ behavior. Despite this, various practical constraints can prevent physical access to the renovated area (e.g., weather conditions, time/resources limits, health issues, bureaucratic constraints). Exploiting digital tools for such an assessment can be a crucial support in such circumstances. The current study presents the visual POE of a restorative garden for older adults in Milan, Italy. We developed a web application, that includes the exp-EIA© patented method, which allows participants to virtually explore a visual simulation of the environment and provide their feedback. We identified 3 representative viewpoints in the redeveloped garden differing from each other for the functions and the design principles that inspired the transformation. For each point of view, we created 360° Virtual Reality photographs, that can be navigated by looking around, i.e., panning, from the standing point of each view. In connection to each virtual scene, a survey was conducted (*N* = 321). The focus was the psychological experience related to each viewpoint, assessed with two psychometric scales investigating the constructs of emotions (pleasure and arousal) and restoration (fascination, being away, coherence, scope, and environmental preference); such information is integrated with behavioral aspects, including the main activities prefigured by participants and their visual exploration of the VR photography. The results of the virtual exploration show that the garden is perceived as restorative, with a more intense effect in a spot purposely designed. The emotions experienced in the garden are positive and a mild level of arousal is observed. The behavioral dimension is characterized by predominantly contemplative activities and contact with nature. A cartographic representation of the psychological and behavioral data is developed, to support the maintenance of the garden.

## Introduction

### Restorative environments

According to the biophilia hypotheses, as a species, we have an inherent affiliation to the natural environment (Wilson, 1984, 2002). Biophilia is “the innate tendency to focus upon life and lifelike forms, and in some instances to affiliate with them emotionally,” namely to feel connected to Nature. Human tendencies to love and take care of Nature are affected by attention, i.e., the ability to focus on natural stimuli effortlessly, actually to be fascinated by Nature (see Kaplan, 1995), and empathy, i.e., to join emotionally to the various life forms, and to participate to their condition. Humans are genetically programmed to function effectively in natural environments and there is evidence for genetically determined biases that affect environmental preference for natural environment (Balling and Falk, 1982). Experimental research has found evidence that restoration from stress and from mental fatigue relates to exposure to Nature (for a review see Berto, 2014). Natural places that allow a shift toward more positively-toned emotional states (for a review see Bratman et al., 2021), positive changes in physiological activity levels, and in behavior and cognitive functioning are called “restorative environments” (Kaplan and Kaplan, 1989; Ohly et al., 2016). The theoretical framework of this research is the Attention Restoration Theory (ART; Kaplan and Kaplan, 1989), which prescribes two distinct types of attention. The first is the directed attention, which is a key ingredient in human performance as it permits to focus on specific tasks despite potential distractions which might arise in daily life. Such type of attention is effortful and can be tiring under certain circumstances (e.g., prolonged mental efforts): mental fatigue indicates that the “inhibitory mechanism” which inhibits distractions, on which direct attention depends on, runs out of energy. The restoration of directed attention requires “an alternative mode of attending that would render directed attention temporarily unnecessary” (Kaplan, 1995, p. 172). Therefore, it is important to find ways to restore the directed attention capacity, and an effective strategy is the exposure to natural environments. In natural environments, a second type of involuntary attention is invoked, hence during these environmental interactions attention is captured in a bottom-up manner and people do not spend energy in suppressing distracting stimuli (Kaplan, 1995; Basu et al., 2019). Natural environments provide a restorative experience not only because of the lack of intrusive stimuli: according to the ART, individual’s benefit comes from the chance to have an experience of “soft fascination,” which is one of the four characteristics of a restorative environment (Kaplan, 1995) and can be described as a moderate fascination accompanied by esthetic pleasure. This featured of natural environments enables not only the recovery of directed attention, but also the opportunity for self-reflection (Herzog et al., 1997). In addition, there are three other components that are likely to contribute to make an environment restorative (Kaplan, 1995): being-away (implies a setting that is distant either physically or conceptually from one’s everyday routine/environment), extent (the environment’s extension in time and space, whether the setting has sufficient coherence and scope to engage the mind and promote exploration), and compatibility (to what degree a setting fits and supports one’s inclination or purpose).

### Designing aging-friendly environments

Access to these spaces is more important for those living in urban areas, in particular for seniors (Sikorska et al., 2020). The older population, aged over 65, is not only more and more increasing but is also a heterogeneous group of older adults with different abilities and needs (Liu et al., 2014). In many countries, following socio-economic, cultural, and political changes, older people have obtained a higher degree of education, better health, and higher incomes. These factors allow them to have more time for leisure, recreational, and learning activities (Yung et al., 2016). As a result, their expectations from outdoor spaces are also changing and concern “active aging,” which pertains to older people’s wishes and needs in terms of integrating physical activities and being outdoor in their daily routines. According to WHO (2007), active aging depends on many factors: some of these are objectives such as physical environment, health and social services, economic conditions, and climate; some others are personal, like behavior, cultural attitude, and social involvement (Sugiyama and Thompson, 2007). This situation confirms the need for more green open spaces in urban areas to promote the well-being and active aging of older people. Restorative gardens serve therapeutic purposes on three levels of physical interaction: active, less active, and passive (Ulrich, 1999). Physical rehabilitation and engaging in horticultural therapy are examples of “active” interaction with the garden. Although studies on horticultural therapy are insufficient due to poor methodological quality, a systematic review on this topic (Kamioka et al., 2014) concludes that this kind of intervention can be an effective treatment for mental and behavioral disorders such as dementia, schizophrenia, depression, and terminal cancer care. On the other hand, restorative gardens are suitable to enhance stress reduction and psychological well-being even through “less active” modes of interactions, as sitting in the garden, observing plants and animals, and listening to nature sound (see Ulrich, 2002 for a review). In a similar fashion, Browning et al. (2019) observed an inverse relationship between depressive symptoms and tree cover surrounding nursing homes. Restorative spaces enhance social interaction and people’s sense of community and safety as well, as the availability of green spaces is important not only for the quality of life of seniors residing in care facilities, but also for the staff and visitors: urban green spaces contribute to physical activities, recreation, and social interactions (Artmann et al., 2017). The benefits of using public green open spaces are well known both on physical and social level. They offer a place for older people to take a break, to connect with Nature and people and for walking, which is the major outdoor physical activity for older people (Yung et al., 2016). The physiological benefits of walking regard the maintenance of physical health and functioning, whereas from a social point of view sense of belonging to a place helps maintaining identity and well-being (Wiles et al., 2009). Rugel et al. (2019) confirmed the association between both measures of accessible neighborhood nature and sense of community belonging as well as a trend in effect sizes, showing how higher values of the accessible neighborhood nature variables were associated with increasing odds of reporting higher levels of sense of community belonging,

Positive effects of natural environments can be further enhanced by designing community resources that can be adapted to the needs of people as they change over time. In such perspective, “aging-friendly” communities offer older citizens the opportunity to engage in activities fostering their own physical and psycho-social well-being. Well-designed gardens can encourage older adults to spend more time actively outdoor (Rodiek, 2010): although outdoor usage is influenced by several aspects, such as weather, health conditions, level of interest, and individual attitudes, it is also strongly related to the characteristics of the physical environment (Rodiek, 2010; Rodiek et al., 2016). Moreover, the presence of restorative properties attenuates attentional fatigue in older people, facilitating many activities that can allow them to be autonomous, including not only attention-related activities but also “memory updating,” i.e., being able to modify schemas to accommodate new input, “environmental memory,” e.g., wayfinding abilities and memory for routes, and “prospective memory,” i.e., memory for future events, together with planning ability (De Beni and Palladino, 2004).

### Research rationale: Visual post occupancy evaluation

This study is a part of a three-year project, named Green Age, developed to design and requalify a small green open space included in a wider community garden (Giardino San Faustino) in the Ortica district in Milan (Italy). The study has been carried out in consecutive phases where two main goals can be recognized: (i) assessment of the current condition, design, and implementation of the garden and (ii) assessment of the benefits for the older people using the redeveloped garden. For the first goal, different groups of users have been involved through 6 focus groups, in order to obtain the most complete representation of needs and expectations of the potential users of the area (Fumagalli et al., 2020; Boffi et al., 2021). After the realization of the garden, in the last part of the project, its use was planned to be evaluated to have feedback of how the requalification supports the requirements of individual end-users through a Post-Occupancy Evaluation (POE).

The POE is a multimethod approach commonly used by environmental psychologists and landscape architects to evaluate built environments in terms of design and users’ reported use and experience (Zimring and Reizenstein, 1980; Zeisel, 1984; Bechtel, 1997). Post occupancy research applied to healing gardens allows identifying the design’s successes and failures, informing designers about possible areas for improvement, and providing specific guidelines for garden maintenance and planning activities (Heath and Gifford, 2001; Sherman et al., 2005; Sidenius et al., 2017; Paraskevopoulou and Kamperi, 2018). Research in this field has focused mainly on the study of healing gardens in hospitals and on optimizing their positive impact on patients, visitors, and staff. In particular, POE enables to evaluate users’ utilization of gardens, determine barriers to use, and investigate perception differences of garden features between users or the impact on their emotional state (Whitehouse et al., 2001; Sherman et al., 2005). A review carried out by Ulrich et al. (2008) has collected a few studies showing how gardens can be an effective restorative setting for stressed patients, families, and staff, fostering an improvement in their emotional well-being. Healing gardens tend to alleviate stress effectively when there is the presence of flowers, water, grassy spaces with trees, spatial openness, and compatible pleasant nature sounds, such as birds and water (Cooper Marcus and Barnes, 1995, 1999; Ulrich, 1999; Rodiek and Schwarz, 2006). The beneficial effects associated with healing gardens placed in healing places, such as hospitals and other therapeutic facilities, suggest considering the same design recommendations for public green open spaces as well. These, enhanced to become true places of healing and reconnection, can become a valuable contribution to health prevention and promotion in the general population (Cooper Marcus and Valente, 2015). Generally, POEs in this field consist of surveys and/or interviews accompanied by more or less structured observations of onsite users’ behavior. Recently Sidenius et al. (2017) have developed a diagnostic POE method for therapeutic gardens focusing on five central examination points:

1. Landscape analysis through observations and operation traces collection with the aim to understand the physical conditions of the environment and establish distinctive location and space.
2. Experience of environment through logbooks or interviews with users with the aim to establish their experiences and their gains from the environment.
3. Operations through observations or interviews focused on the use and activities done in the garden.
4. Experience of operations through observations or interviews finalized to establish users’ experiences of their use of the garden.
5. Health and wellbeing outcomes through questionnaires, logbooks, or interviews.

A recent review (Paraskevopoulou and Kamperi, 2018) showed that post-occupancy research design recommendations can vary among users. The review summarized the main evidence-based design recommendations for healing gardens identified for each kind of user: children in pediatric hospitals, cancer patients, nurses, and so on. A study about older residents in assisted living facilities reported which features of the physical environment tend to influence their use of outdoor green areas (Rodiek, 2003). An abundance of walkways to access outdoor landscaped areas, presence of shade and seating along the walkways, abundance of vegetation, access to views looking beyond the facility boundaries, presence of windows adjacent to outdoor entries, and areas near entries for previewing outdoor spaces are all important features encouraging older people outdoor usage.

Considering the lack of specific design guidelines for restorative gardens in open public contexts and the difficulty of integrating in the design proposal different types of users, we assigned key importance to the involvement of older people also in the post-occupancy evaluation phase, as they were already engaged during the co-design phase to support the inclusion of their needs in the design project (Fumagalli et al., 2020; Boffi et al., 2021). Despite this, on-site involvement of people was greatly challenged by the COVID-19 emergency and by the need to respect social distances. This is especially true for older people, whose use of public and open spaces was particularly affected by the health risks resulting from the pandemic period. These contextual constraints required a review of the assessment method, from on-site to online; such an approach is supported by a growing literature investigating how beneficial effects of natural environments can be observed also through different forms of environmental simulation (Yeo et al., 2020; Browning et al., 2021; Pasca et al., 2021). Despite the promising results, it is still unclear to what extent the psychological reactions to simulated natural environments can be considered comparable to those observed in physical natural environments. Calogiuri et al. (2018) have reported similar restorative effects after a walk in physical and simulated environments, even if the latter are affectively less enjoyable. Another experimental study has shown that nature exposure can have restorative and positive mood effects both in outdoor environment and its 360-degree virtual reality video counterpart compared to an indoor control group, despite the increase in positive mood is observed only in the outdoor condition (Browning et al., 2020a). According to Reese et al. (2022) the increase of positive and reduction of negative affect after forest bathing takes place both in a physical and simulated environment, despite a larger size effect in the physical environment. Consistently, Chirico and Gaggioli (2019) found similar emotional responses when exploring a natural landscape in real-life and through 360-degree videos. Notwithstanding the differences of various studies, a meta-analysis has indicated that only actual natural environments can increase positive affect, whereas the reduction of negative affect can be observed also in the simulated settings (Browning et al., 2020b). Even if a final answer about virtual and physical comparability is not yet available, current data suggest a stable emotional reaction to virtual reality simulations beyond age and familiarity with technology (e.g., Browning et al., 2020a; Vahle et al., 2022). In addition to the reflections on the psychological reactions, many efforts have been made to determine the type of simulation required for a reliable experience. A recent review has shown that two-thirds of the studies on the psychological effects of simulated natural environments only rely on visual stimuli, which is the only sensory channel always present in all types of simulation included in the review (Browning et al., 2021); despite this, sound plays a key role in environmental simulations, as it is comprised in more than one-third of the studies examined by Browning et al., and soundscape is specifically investigated in studies on the effects of different types of environment (see Dubois et al., 2006; Krzywicka and Byrka, 2017, 2020). Browning et al. (2021) have also suggested that despite “multisensory simulations may provide untapped research opportunities […] few of these methodological decisions influenced study findings” (p. 710). However, studies on the quality of soundscape have shown that the interaction between road traffic and natural sound is a crucial element (Axelsson et al., 2010), especially when assessing the perceived quality of soundscape in quiet areas (Nilsson and Berglund, 2006). In addition, Browning et al. (2021) have pointed out that more immersive simulations are not more likely to induce positive effects, which may be elicited by still images only. Yet, we highlight that manipulatable images are a valuable resource not only for simulation purposes but also for data collection, as they make possible the integration of behavioral information about the interaction with the environment. All those reflections must be carefully considered when assessing the effort required for an environmental simulation (Brivio et al., 2021). Hence, for the purposes of the current study, we define a visual POE as an evaluation of a regenerated/newly built environment taking place after the physical intervention is completed but before full onsite access is possible, occurring through a visual-only exploration of the environment. The aim of this study is carrying out a visual POE to assess the restorative effect on elders and on a larger sample of a restorative garden, designed for older people’s benefit. We apply a methodology that allows us to investigate four of the five central examination points indicated by Sidenius et al. (2017) even without physical access to the site and exploiting virtual reality photography (Highton, 2010; Benoit et al., 2015): establishing distinctive locations in the garden (limited to visual exploration behavior); the visual experience of the environment, with particular attention to the possibility of living a restorative experience in it and related emotions; the operations elicited by the visual experience, namely the use and activities that participants expect to do in the garden; reflecting on health outcomes from visual exposure, relying on the data from restoration and emotions.

## Materials and methods

### The study area

**Figure 1:**
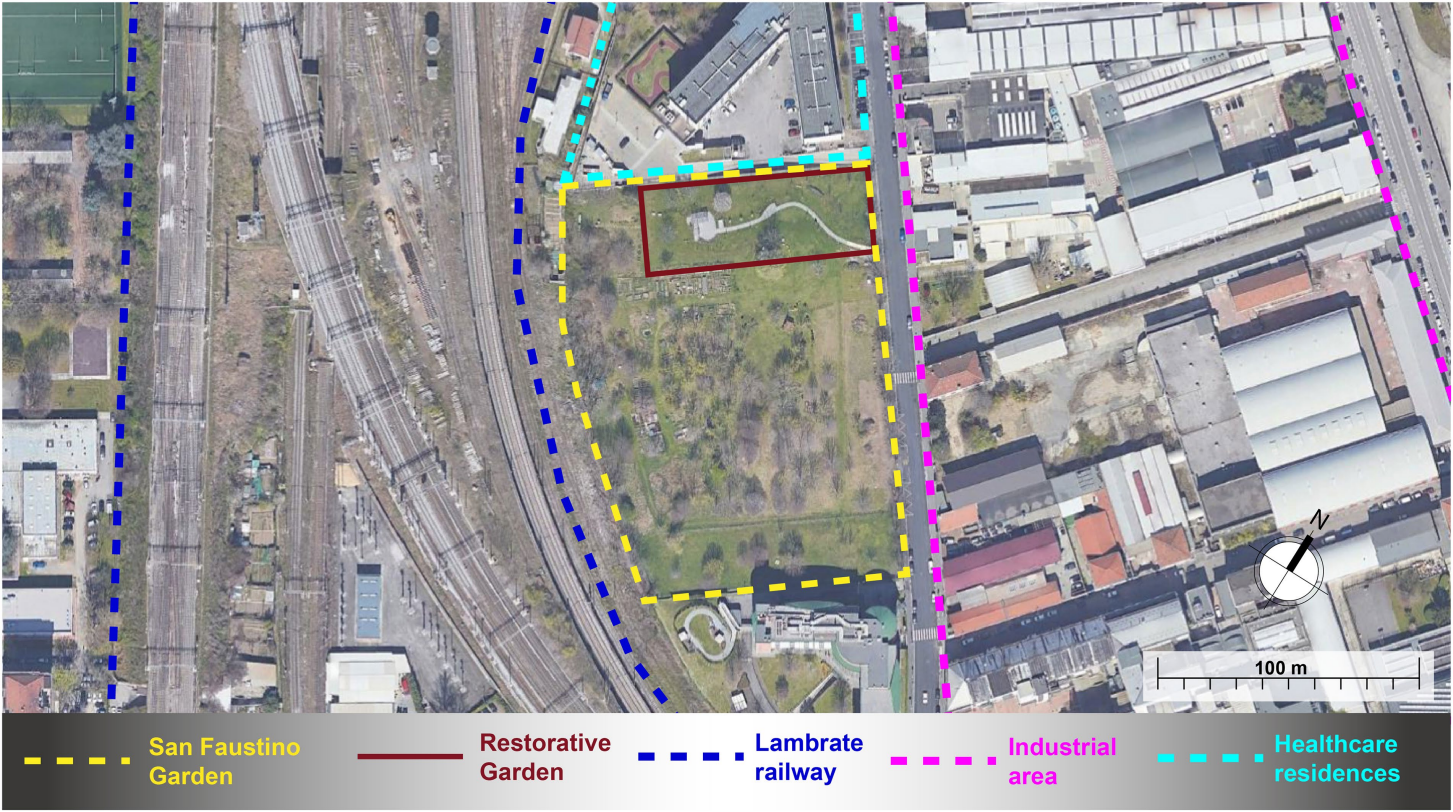
The overview of the area surrounding the restorative garden.

**Figure 2:**
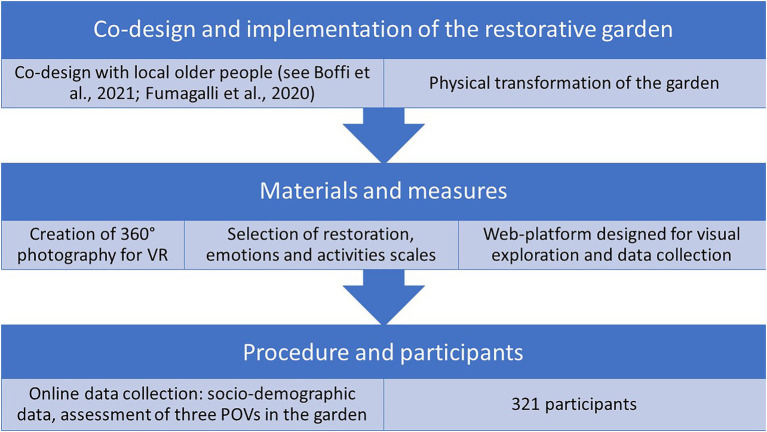
Research framework diagram.

The restorative garden of the Green Age project is developed within the larger San Faustino Garden, a community garden regenerated thanks to the interest of some associations in the Ortica district (Figure 1). Owned by the University of Milan and given on loan to Town Hall 3 of Milan municipality, San Faustino Garden has become one of the largest community gardens in the city, located in a once industrial district now increasingly residential and close to the railway ring in the eastern part of the city. Being surrounded by several infrastructures, the garden is affected by varying levels of noise pollution (55–59 dBA in most peripheral areas of the garden, 50–54 dBA in the inner parts) by the railway and the closest road; the surrounding industrial areas have a sound immission limit up to 70 dBA; however, no empirical data are available to assess the actual effect of the semi-abandoned industrial area on the garden (Municipality of Milan, 2021). Inside the community garden, the area of the Green Age project (2,700 m^2^) is bounded to the North by the plot of a Social Assistance Residence (RSA) for older people, to the East by via San Faustino (medium traffic road), to the West by the elevated railway that separates it from the central districts of the city, to the South the view is open toward the Community garden, with vegetable gardens, fruit plants, and spontaneous trees. The project area, before the construction of the restorative garden, was basically a large lawn area devoid of vegetation, if not for a large cherry tree and some spontaneous maple trees. The designers of the restorative garden are part of the research team (see “Author contributions”) and share a background in biophilic design as researchers and professionals. The financing of the project foresees both the co-design of a restorative garden, its implementation, and the assessment of the psychological effects (Figure 2).

### The restorative garden

**Figure 3:**
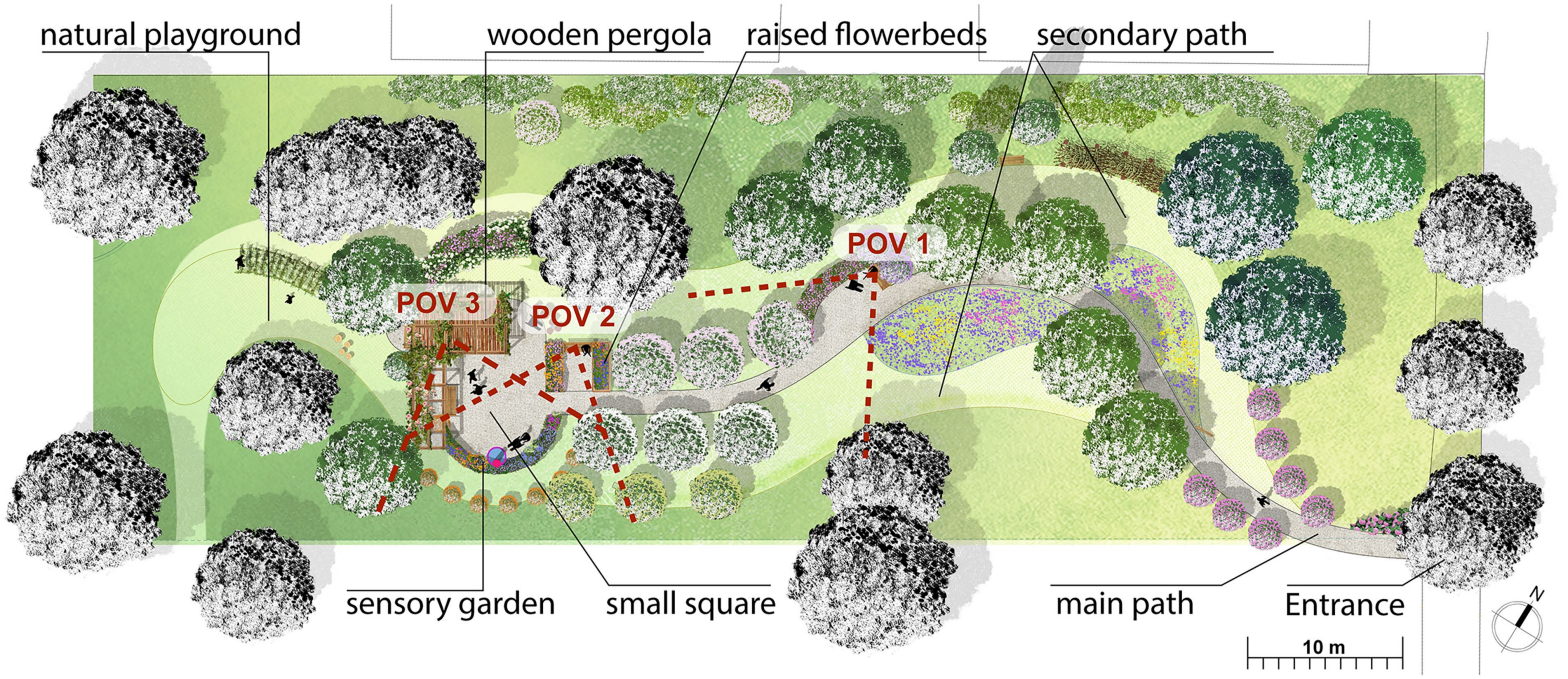
The design project plan of the restorative garden.

The area (Figure 3) is accessed from via San Faustino through different blooms as a welcome sign communicating what takes place inside. To facilitate the access for wheelchairs, a pedestrian gate and a small section of concrete path are set. The focal area of the design project is a small square where the main activities are concentrated: it is quite far from the road, close to the existing trees that create a niche for protection and a sense of embracement. The design components that define a restorative garden project belong to different disciplines: landscape architecture, medical sciences, environmental psychology, and landscape ecology. The result of their interaction is a prosthetic environment, in which nature is a partner in the treatment process, with ecological, landscape, and cultural values (capable of providing ecosystem services). Design criteria for public restorative gardens, which could enhance the quality of older people life, can be summarized in three main points: prosthetic environment, regenerative place, and Ecosystem value (see Fumagalli et al., 2020 for a complete review of applied design criteria and their integration in a co-design process). Despite the needs of older people being taken into account both among the design criteria and during the participatory process, the universal design approach is applied (Connell et al., 1997; Story, 2011) to identify design solutions that are suitable for the greatest population possible. This is intended to favor intergenerational social activities and provide a resource that is valuable for the whole neighborhood, instead of being limited to the host of the nursing homes.

The restorative garden is crossed by two paths, a main one in permeable paving (1.5 m wide) and a secondary one for “exploration” on cut lawn, to allow guests to explore the garden according to their physical abilities and moods. The main one passes through a group of mulberry trees (typical trees of the Lombard countryside), an orchard (designed with the intention to be a catalyst of historical memories and symbol of prosperity and fertility), and two raised flowerbeds with edible flowers, designed in compliance with anthropometric measures (accessible by those in wheelchair) and surrounding a wooden bench; the main path leads to a small square and to a wooden pergola, with climbing roses, vines, and honeysuckles, designed to stop, chat, and do community activities. The area is provided with movable furniture, tables and chairs, and fixed wooden benches. The square is framed to the South by a sensory garden, with aromatic shrubs and perennials, particularly attractive species for pollinating insects and a colored tube fountain in the middle. To the East, a bird garden with feeders (designed to facilitate interaction with small animals) was built with trunk sections and adorned with small silhouettes of blackbirds. Beyond the small square, clearly visible from the pergola, a small natural playground for children is designed with a willow tunnel and wooden play sculptures. Halfway along the main path, a rest area with a wooden bench is set for those who want to rest and resume their journey, or turn back (round trip-walk). The secondary path has been designed to enhance more individual experiences, in more “naturalistic” areas among flowery meadows, Benje’s hedge (dead hedge), typical trees of the lowland forest, and faunistic hedges with edible fruits. Along both paths, the view toward the South remains open to the garden and the surrounding landscape; a naturalistic hedge was created along the northern border, with the function of shielding the large, particularly impactful building of the nearby RSA. The aim is to create a gradual psychological separation from the nursing home. Composed by small native trees and shrubs it serves as habitat and food reserve for mammals, pollinators, and beneficial insects and facilitates movement through the garden for animals.

### Materials and measures

Data collection is carried out with an original web platform designed by the authors (see Acknowledgments) which enables both the virtual exploration of the restorative garden through 360° pictures from pre-set points of view and the data collection. The authors identified three representative Points of View (POVs; Figure 3) consistently with the restorative garden’s features and the main functions foreseen by the landscape design project; the criteria for selection took into account the design goals of favoring contact with nature and social relationships for older people (see Fumagalli et al., 2020; Boffi et al., 2021), thus seating areas are considered as the key places for assessment. POV 1 is placed at the wooden bench in the rest area, which is located near the entrance and along the main path. This bench faces the core of the design project from a distance; indeed, from this perspective, the atmosphere of the core area is already disclosed and it is possible to foresee the experience of the central small square and its sensory garden. This spot is meant to keep together natural and social goals. POV 2 is adjacent to the small square and placed at the wooden bench between the raised flowerbeds. This location is designed for providing an experience of immersion into the natural environment, as the raised flowerbeds visually emphasize the presence of flowers, favoring other sensory experiences (e.g., olfactive and tactile) also for those on a wheelchair. POV 3 is placed under the pergola, an area explicitly designed as an attractor of social activities; indeed, a table and some chairs (not present in the panorama) are foreseen under the comfortable shadow of the wooden pergola. By looking around from this perspective almost all the redeveloped area is visible. A 360° picture is taken at each POV at the eye level of a person sitting on a bench/wheelchair, to create three spherical panoramas that can be freely explored panning the picture by clicking and dragging on a pc or tapping the screen on a mobile device. The visual exploration allows participants to change the orientation of the camera corresponding to the position of the head in space, with a rotation about three perpendicular axes: yaw (normal axis), pitch (transverse axis), and roll (longitudinal axis); the location of the observer in each POV corresponding to the position of the body in the geographical space is stationary. For each POV a starting direction for the visual exploration is set, aiming at the focal area represented by the small square according to the same criteria adopted for the POVs. The 360° pictures are rendered and embedded with Marzipano plugin,^1^ whose data are dynamically collected in the questionnaire through a script designed by the authors. The pictures were taken on April 17, 2021, on a sunny day; movable furniture (tables and chairs under the pergola) is absent in the pictures, whereas fixed wooden benches are already in place.

Before starting the exploration, the data collected include socio-demographic variables regarding the characteristics of the participants (gender; age). Before exploring each POV, general instructions ask the participants to explore the place freely and, when a viewpoint draws their attention, stop the exploration on that point and start answering the following questions. The effects of each POV are investigated through three tools measuring restoration, emotions, and activities associated with the environment.

- Restoration is assessed using the Italian version of the Perceived Restorativeness Scale-children (PRS-ch; Berto et al., 2015), measuring the individual perception of the restorative value of a place. The scale comprises 18 items related to the factors being away (e.g., “In this place I do not think at my worries”), fascination (e.g., “This place is interesting”), coherence (e.g., “In this place it is easy to see what’s around me”), scope (e.g., “In this place I am free to play, run and move”), and environmental preference (e.g., “I like this place”). This version is chosen as the wording is conceived to be more comprehensible and hence is more suitable for use with older people, while it respects the original principles of ART (Kaplan, 1995) and the content of the item is consistent with the adult version of the PRS (Purcell et al., 2001; Pasini et al., 2009). Judgments are made on a 5-points Likert scale (1 = “not at all,” 5 = “very much”), and the final score is given by the mean value of the 18 items.
- The emotions are assessed with an original native digital scale which represents an evolution of the Self-Assessment Manikin (SAM; Bradley and Lang, 1994), a widely used self-reporting tool measuring the dimensions of pleasure and arousal. Such dimensions represent how pleasant/unpleasant and activating/deactivating an emotion is perceived, and their combined values provide a comprehensive description of a person’s affective state, which can be investigated in association with specific stimuli, like a place in the current study. This conception of emotions is consistent with the circumplex model described by Russel (Russell and Pratt, 1980), and the values of pleasure and arousal provided by each participant can be conceived as coordinates of a Cartesian plane where each position corresponds to a specific affective state defined by univocal labels (qualitative feature of the emotion) and intensity (quantitative feature of the emotion). Following the approach adopted in developing the affective slider (Betella and Verschure, 2016), we designed a fully pictorial tool with two slider controls, including icons placed at the ends of the two slider controls to represent the continuum of pleasure and arousal. The participants click on each slider control moving the cursor placed at the center, data are collected by transforming the position of the cursor into the corresponding value.
- Activities are collected through a list of possible activities belonging to six different categories: creative activities (e.g., “drawing”), contemplative activities (e.g., “resting”), interaction with nature (e.g., “observing wild animals”), social interactions (e.g., “socializing”), fitness (e.g., “physical exercise”), and break (e.g., “eat”). The activities are presented without categorization, and participants can select up to three activities for each POV.

### Procedure and participants

The landing page of the questionnaire contains a presentation of the research project and an informed consent which, once accepted, directs participants to the socio-demographic form. Subsequently, a brief instruction page explains the functioning of the tool for the visual exploration of the garden, including a map showing the location of the POVs. The POVs are presented in a random order, and during the visual exploration participants are invited to click on a small button superimposed to the picture when a viewpoint draws their attention. By doing this, they terminate the exploration and start answering the questionnaire enabling the data collection of the psychological variables related to the single POV. Once the questionnaire is completed, the following POV is presented and the procedure repeats until the completion of all three POVs. The study is conducted in accordance with the Declaration of Helsinki, and the protocol is approved by the Ethics Committee of University of Milan (Project Green age. Green Space for Active Living. Older Adults perspectives) on April 19, 2019.

The invitation to perform the digital exploration and fill in the questionnaire is distributed with the link to the questionnaire via mailing lists and webpages related to associations and informal groups active in the Ortica neighborhood and the surroundings, taking advantage of previous contacts gathered during the co-design phase. A total of 321 participants completed the activity (age *M* = 41.14, s.d. = 21.49, Min = 16, Max = 93; 63% female, 37% male), and 110 evaluated all the POVs.

### Analyses

Visual exploration behavior is analyzed according to the exp-EIA© method (Experiential Environmental Impact Assessment method), which considers both the position of the observer, corresponding to the three POVs in the current study, and the target point, corresponding to the final direction of the sight resulting from the orientation of the virtual camera when a participant chooses a portion of the visual landscape for the assessment (no information about the continuous movement of the camera is considered in this study). The method clusters the participants using the DBSCAN method (Birant and Kut, 2007) with Scikit-learn 0.22 and Python 3.8 libraries, hence identifying groups of participants that share a similar visual exploration behavior: each cluster includes people who are in the same position and look in a similar direction; the method recreates the average geographical position and visual target for each cluster (*N* = 321). This information is the basis for representing a partial isovist, i.e., the portion of space visible from a specific point of view and with a single target (Benedikt, 1979), associated not with a single observer but with a cluster, that we define as “clustered isovist” (Patent for Invention application no. 102021000017168–30 June 2021; International application N. PCT/IB2022/055823 - 23 June 2022). The clustered isovists are represented on maps combining Open Street Map data and the plan of the designed restorative garden.

According to the exp-EIA© method, the visual exploration behavior is combined with the collected psychological data, which are analyzed in three ways. Relying on analyses described in the previous paragraph, descriptive statistics are used to represent restoration, emotions, and activities associated with each cluster, including the clusters positioning on the emotional Cartesian plane. Subsequently, restoration and emotions are integrated with geographical information, producing clustered isovist containing psychological information (Piga et al., 2021a). Finally, inferential statistics are applied using statistics software (IBM SPSS Statistics, V.27) to identify significant differences. Due to the reduced number of observations in some clusters, these analyses are limited to the most numerous ones. One-way repeated-measures ANOVAs are conducted to determine whether there are statistically significant differences within-group (*n* = 71) in restoration, pleasure, and arousal. A Pearson’s correlation coefficient is computed to assess the linear relationship between restoration, pleasure, and arousal separately evaluating the clusters. Analyses considering significant differences according to age and frequentation of the community garden are applied to POVs without differentiating among clusters, due to the scarcity of observation that would result combining all these selection criteria. Comparisons between activities selected by over and under 60 years old are performed using Fisher-Exact or the Chi-squared test where applicable (*n* ≥ 5). Differences in restoration, arousal, and pleasure between over and under 60 years old and between people who have visited the community garden and not are performed using Student’s *t*-tests. The results are considered significant when the *p*-value is <0.05 (Sirkin, 2006).

## Results

### Visual exploration behavior map

**Figure 4:**
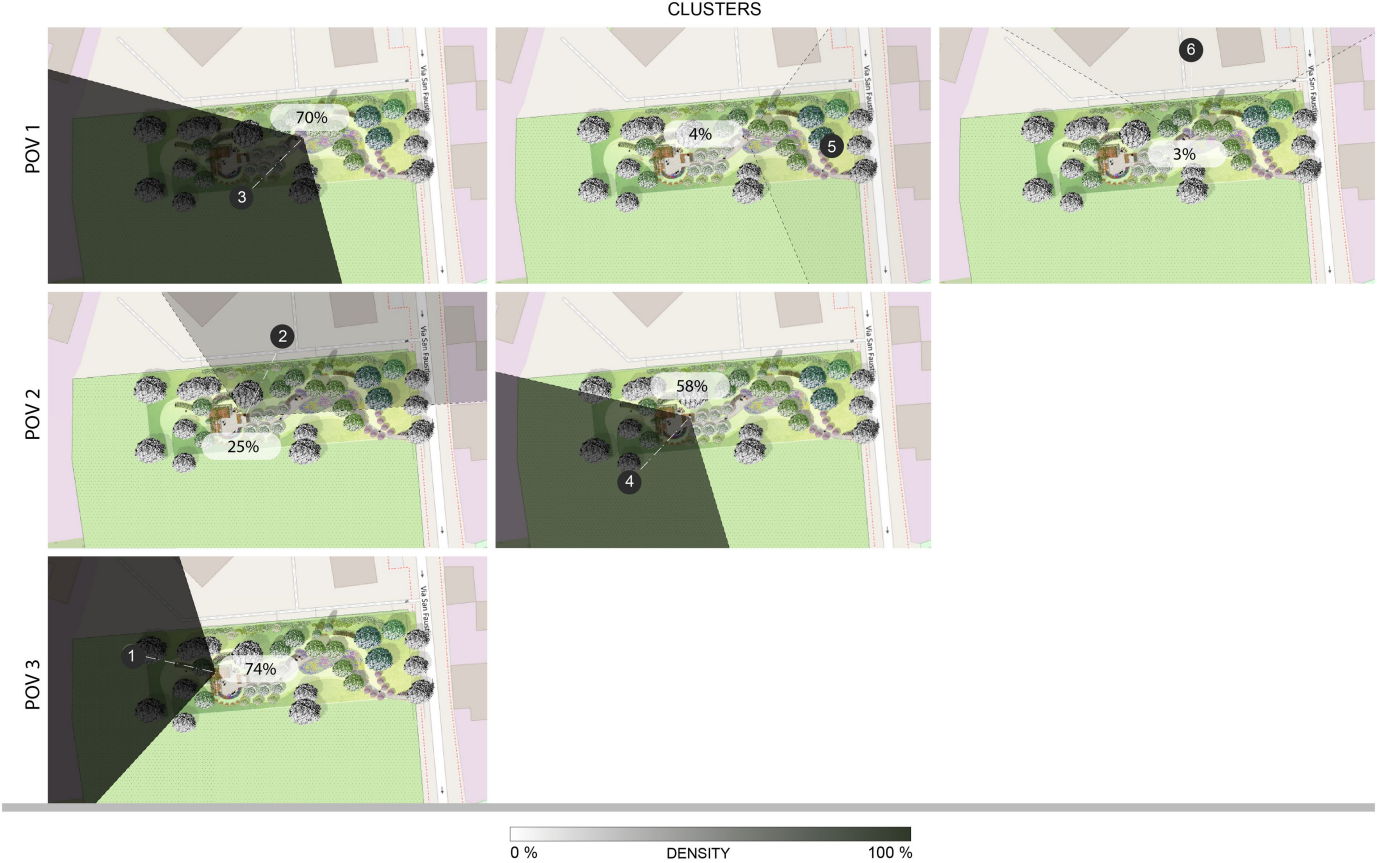
The restorative garden, with the isovists associated to the clusters identified in each POV: they describe the visual exploration behavior. POV 1 – the bench on the path, clusters 3, 5, and 6; POV 2 – the bench between the flowerbeds, clusters 2 and 4; POV 3 – pergola, cluster 1.

The cluster analysis, which excludes the outliers, identifies 6 clusters for the 3 POVs (Figure 4), that show the sections of the environment which mainly attracted the attention of the observers. The number of participants included in each cluster varies: this information is represented in Figure 4 through opacity (the opaquer the isovist, the higher the number of people included in it; a label specifies the percentage of participants in each cluster), a reflection on such information is developed in clusters and age comparison: restoration, emotions, and prefigured activities. POV 1, corresponding to the bench on the main path, gives origin to 3 clusters: cluster 3, oriented toward South-West where the orchard is located (*n* = 133; age *M* = 40.6); cluster 5, oriented toward East where the mulberry trees and the entrance of the garden are located and the external urban environment is visible beyond the fence of the garden (*n* = 7; age *M* = 45); cluster 6, oriented toward North where the naturalistic hedge and the RSA building are located (*n* = 5; age *M* = 43.4). POV 2, corresponding to the bench between the flowerbeds, gives origin to 2 clusters: cluster 2, oriented toward North-East where the naturalistic hedge and the RSA building are located (*n* = 46; age *M* = 43.5); cluster 4, oriented toward South-West where the small square is located, specifically aiming at the sensory garden and the colored fountain (*n* = 111; age *M* = 41.3). POV 3, corresponding to a position under the pergola, gives origin to cluster 1, oriented toward the West where the playground for children is located (*n* = 141; age *M* = 41.8).

### Restoration chart and map

**Figure 5:**
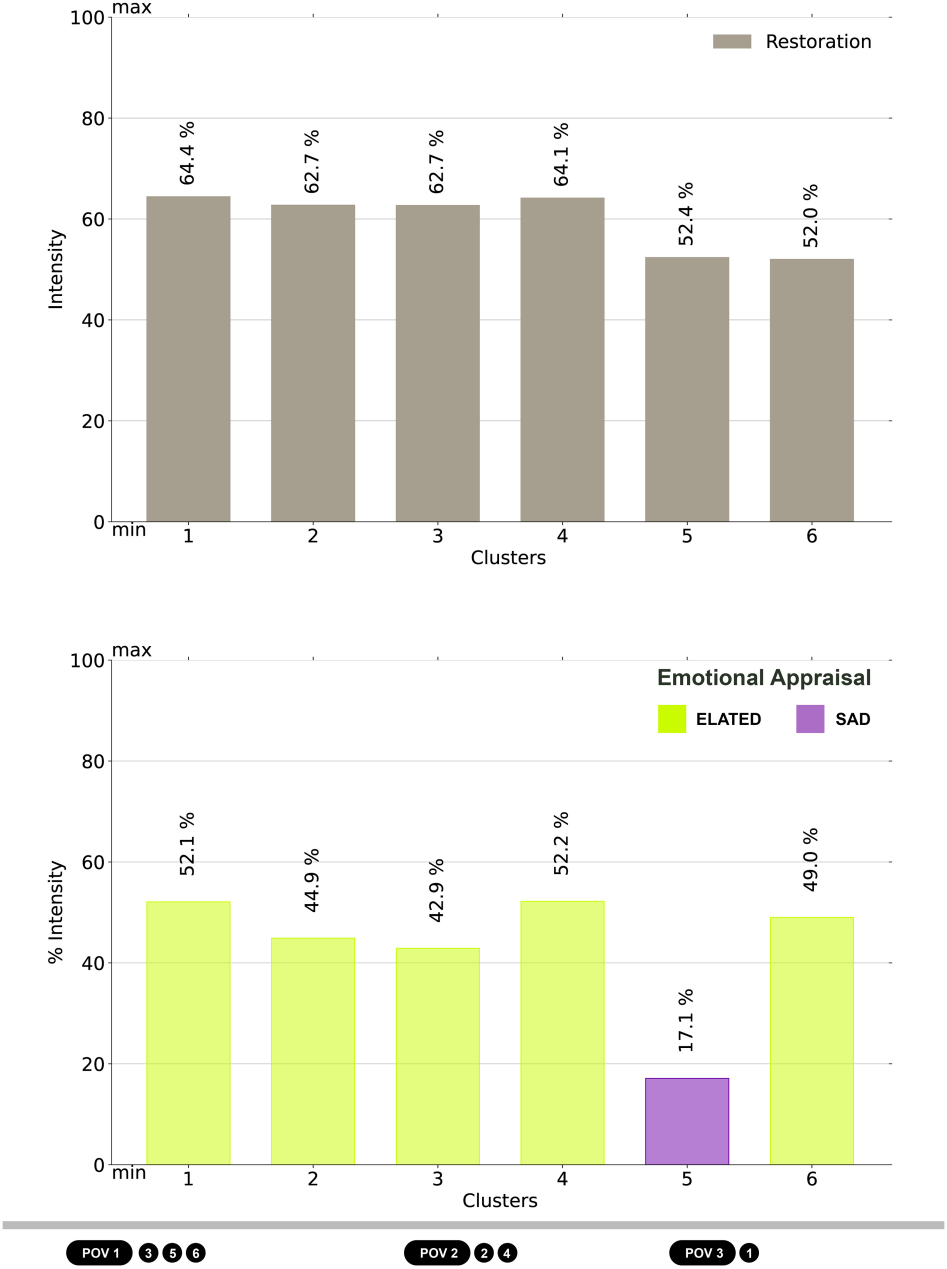
Charts of restoration (top, corresponding to the mean value of the restoration scale) and emotions (bottom, corresponding to the position on the Cartesian plane of the circumplex model obtained with the values of pleasure and arousal; the quality of the emotion is determined by the segment of the plane, the quantity of the emotion is determined by the distance from the center of the plane, the color is determined by the position of the cluster on the plane. See Figure 7 for details) calculated for each cluster: POV 1 – the bench on the path, clusters 3, 5, and 6; POV 2 – the bench between the flowerbeds, clusters 2 and 4; POV 3 – pergola, cluster 1.

The PRS-ch shows good reliability (Cronbach’s Alpha = 0.93). The levels of restoration resulting from the scale are transformed into percentages and are above the middle value for all observed clusters (Figure 5, top). In particular, clusters from 1 to 4 show higher values between 62.7 and 64.4%, whereas clusters 5 and 6 remain below the threshold of 52.4%.

**Figure 6:**
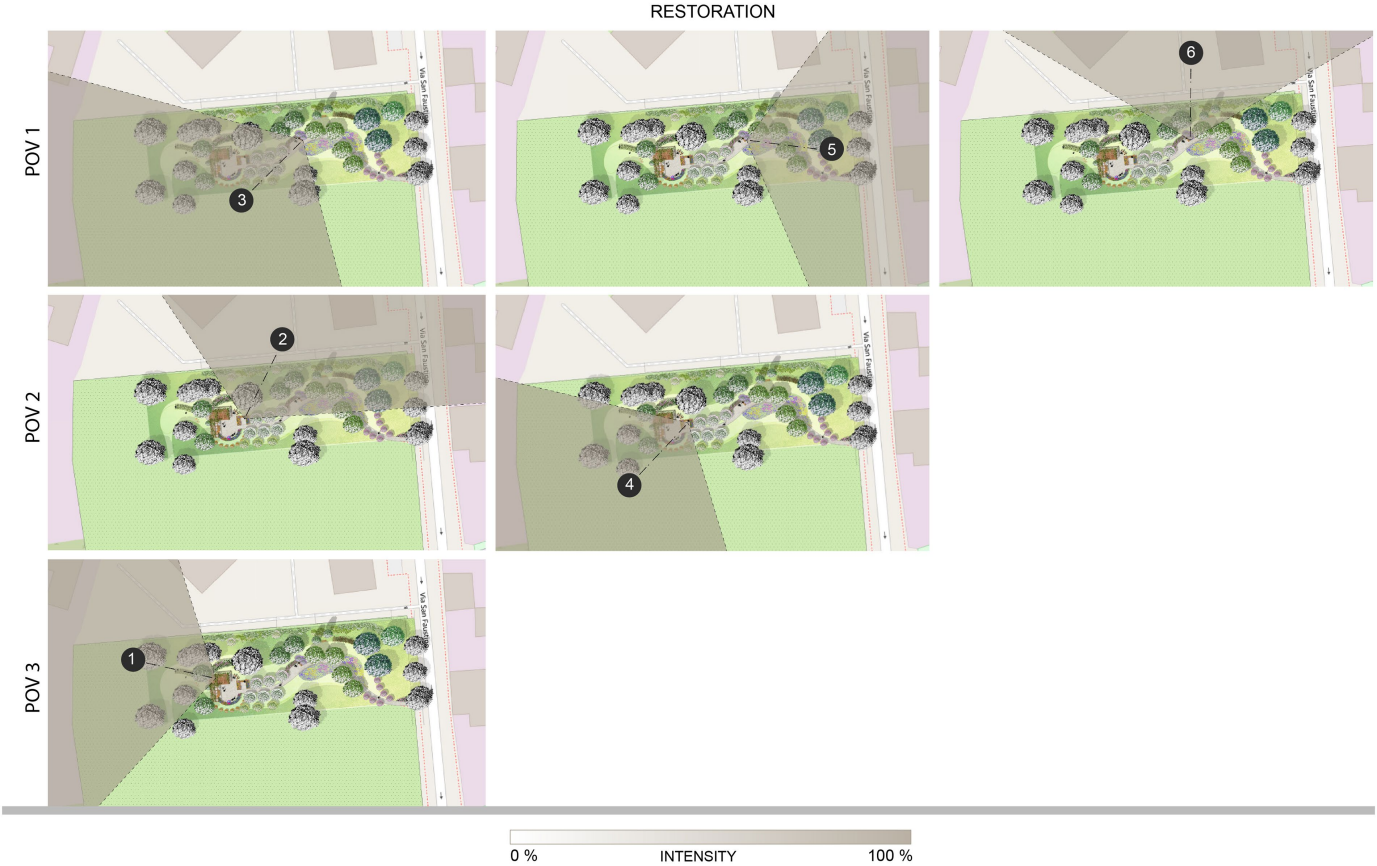
The restorative garden, with the isovists associated to the clusters identified in each POV colored according to the intensity of the restoration: they integrate the visual exploration behavior and the restorative effect. POV 1 – the bench on the path, clusters 3, 5, and 6; POV 2 – the bench between the flowerbeds, clusters 2 and 4; POV 3 – pergola, cluster 1.

The maps representing the restoration clustered isovists (Figure 6) highlight that the clusters oriented toward the restorative garden and its natural elements are associated with higher values of restoration (e.g., clusters 1, 3, and 4), whereas those including more built and urban elements show a decrease around 10% (cluster 5, toward the urban context; cluster 6, facing the RSA building). An intermediate condition is observed for cluster 2, which combines a high level of restoration despite being oriented toward the RSA building. It must be observed that in this case the portion of visible environment also comprises a tall tree in the foreground for the observer (see Figure 6, map middle-left), which can emphasize the effect of the naturalistic hedge compared to cluster 6, where the hedge is accompanied by smaller trees and shrubs.

### Emotions chart and map

**Figure 7:**
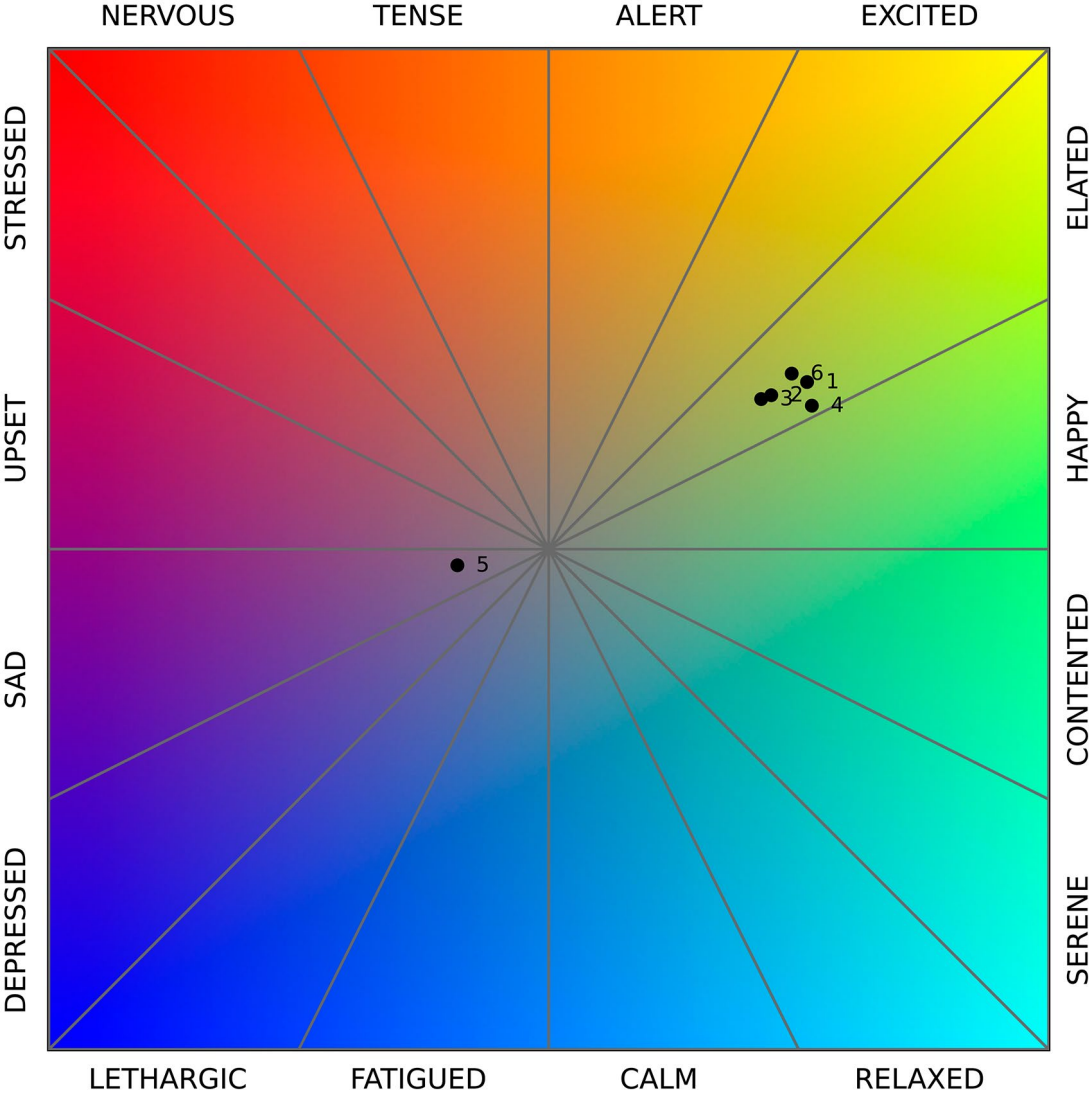
Mean values of pleasure (*x*-axis) and arousal (*y*-axis) for each cluster, positioned on the Cartesian plane described by the circumplex model: POV 1 – the bench on the path, clusters 3, 5, and 6; POV 2 – the bench between the flowerbeds, clusters 2 and 4; POV 3 – pergola, cluster 1. Source: chart based on Russell’s circumplex model, elaboration by the authors.

The circumplex model describes a Cartesian plane, where moving from the left to the right we encounter emotions changing from unpleasant to pleasant, and from the top to the bottom emotions passing from activation to deactivation. The combination of these two axes, corresponding to pleasure and arousal, results in 16 segments defining the emotions distributed on the plane. Even when included in the same segment, hence being associated with the same emotion, two points can differ in terms of intensity: the closer to the center of the plane, the lesser intense the emotion. In Figure 7 each dot shows the average emotional reaction of a cluster. All clusters except for cluster 5 are in the “elated” segment, in a middle position of intensity: they are connected to a pleasant and moderately activating emotional state. Cluster 5 exhibits almost opposite features, as it is characterized by a slightly deactivating and unpleasant emotional reaction, which can be labeled as lightly “sad.”

The representation on a chart of the emotions associated with clusters (Figure 5, bottom) enables a better quantification of the intensity, which emphasizes commonalities and differences among the clusters. Clusters 1, 4, and 6 share similar levels of intensity, ranging from 52.2.% to 49, whereas clusters 2 and 3 are slightly lesser intense (44.9 and 42.9%). The negative emotional state of cluster 5 is partially compensated by its low intensity (17.1%).

**Figure 8:**
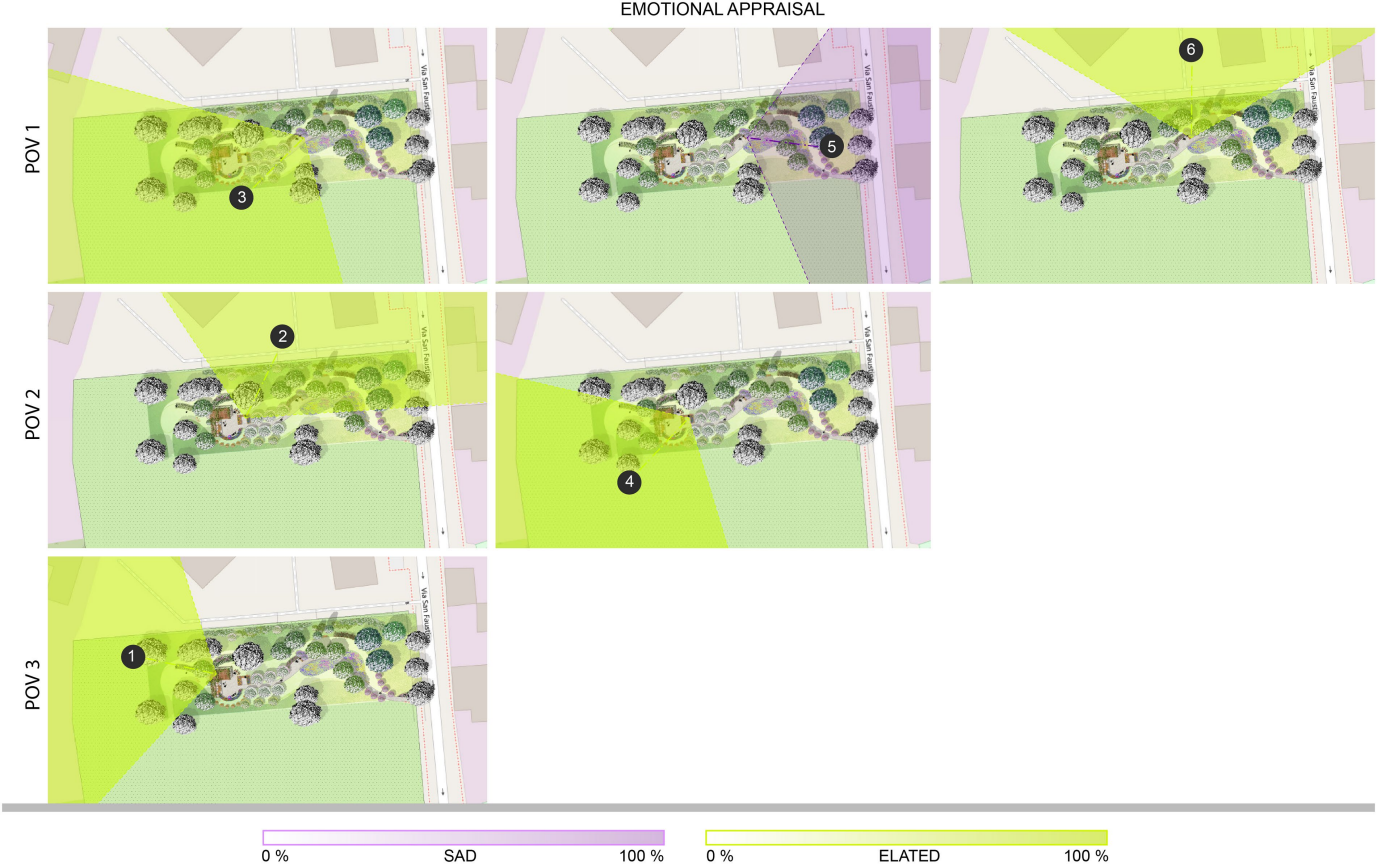
The restorative garden, with the isovists associated to the clusters identified in each POV colored according to the emotions (consistently with the position on the Cartesian plane of the circumplex model, see Figure 7): they integrate the visual exploration behavior and the emotional state. POV 1 – the bench on the path, clusters 3, 5, and 6; POV 2 – the bench between the flowerbeds, clusters 2 and 4; POV 3 – pergola, cluster 1.

The maps representing the emotion clustered isovists (Figure 8) show a pattern partially similar to the restoration maps. All the clusters facing predominantly natural elements result in an elated state (clusters 1, 3, and 4), yet also those oriented toward the RSA building (clusters 2 and 6) share the same emotion. In addition, the values of intensity do not consistently decrease in the presence of the building compared to other clusters. Cluster 5 is the only one including the urban context, which is characterized by an industrial area partially abandoned: the lower performance observed on restoration values is confirmed also by this representation of emotions, emphasizing the association of this portion of the environment with sadness.

### Clusters and age comparison: Restoration, emotions, and prefigured activities

**Figure 9:**
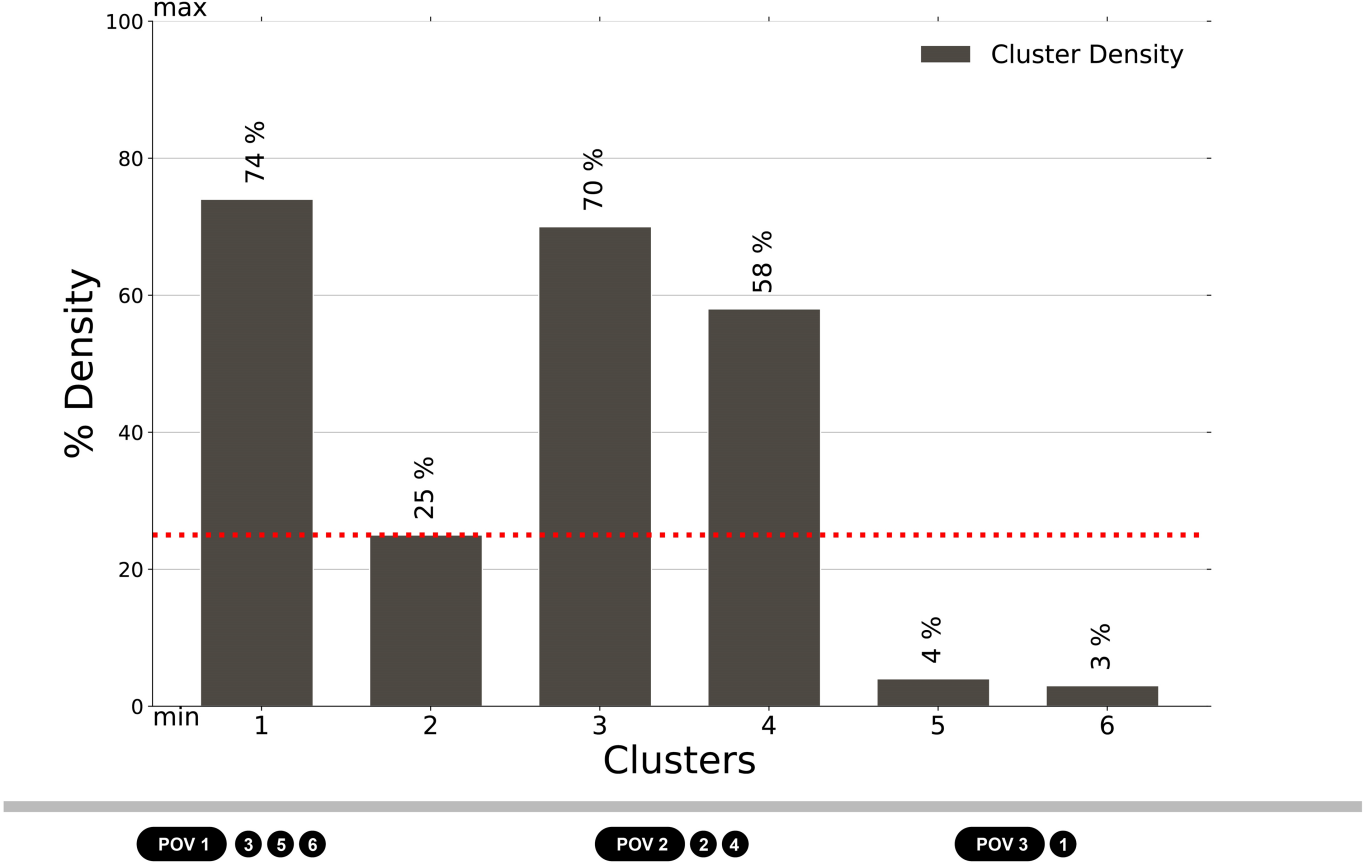
The cluster density, showing the percentage of respondents who were included in each cluster according to their visual exploration behavior: POV 1 – the bench on the path, clusters 3, 5, and 6; POV 2 – the bench between the flowerbeds, clusters 2 and 4; POV 3 – pergola, cluster 1. The most numerous clusters are homogenously distributed across the three POVs (POV 1 – the bench on the path, cluster 3; POV 2 – the bench between the flowerbeds, cluster 4; POV 3 – pergola, cluster 1).

In order to integrate previous descriptive statistics with inferential statistics, we carried out a closer examination of the dimension of the sample for each cluster. Figure 9 shows cluster density, that is, the percentage of people assigned to each cluster among the total visual explorations recorded for each POV, excluding those participants whose behavior is classified as an outlier. Cluster 1 represents 74% of the participants who explored POV 3, being the most populated cluster and the only one associated with POV 3. Cluster 3 is the second largest cluster, including 70% of the explorations of POV 1; clusters 5 and 6 belong to the same POV, representing, respectively, 4 and 3% of the total. Finally, Cluster 4 is the third most populated cluster with 58% of the explorations of POV 2, which is also represented by cluster 2 which contains 25% of the participants. Taking into account that the three largest clusters are distributed across the three POVs, and that the biggest decrease in sample size is observed between cluster 4 and cluster 2, we aim to prevent an excessive loss of cases due to missing values in a within-group analysis. Hence, we consider the first three clusters as adequately representative of the POVs (cluster 1 for POV 3, under the pergola; cluster 3 for POV 1, on the bench on the path; cluster 4 for POV 2, on the bench between the flowerbeds. Above the red line in Figure 9) and carry out the inferential statistics on those three excluding the others from further analyses.

**Figure 10:**
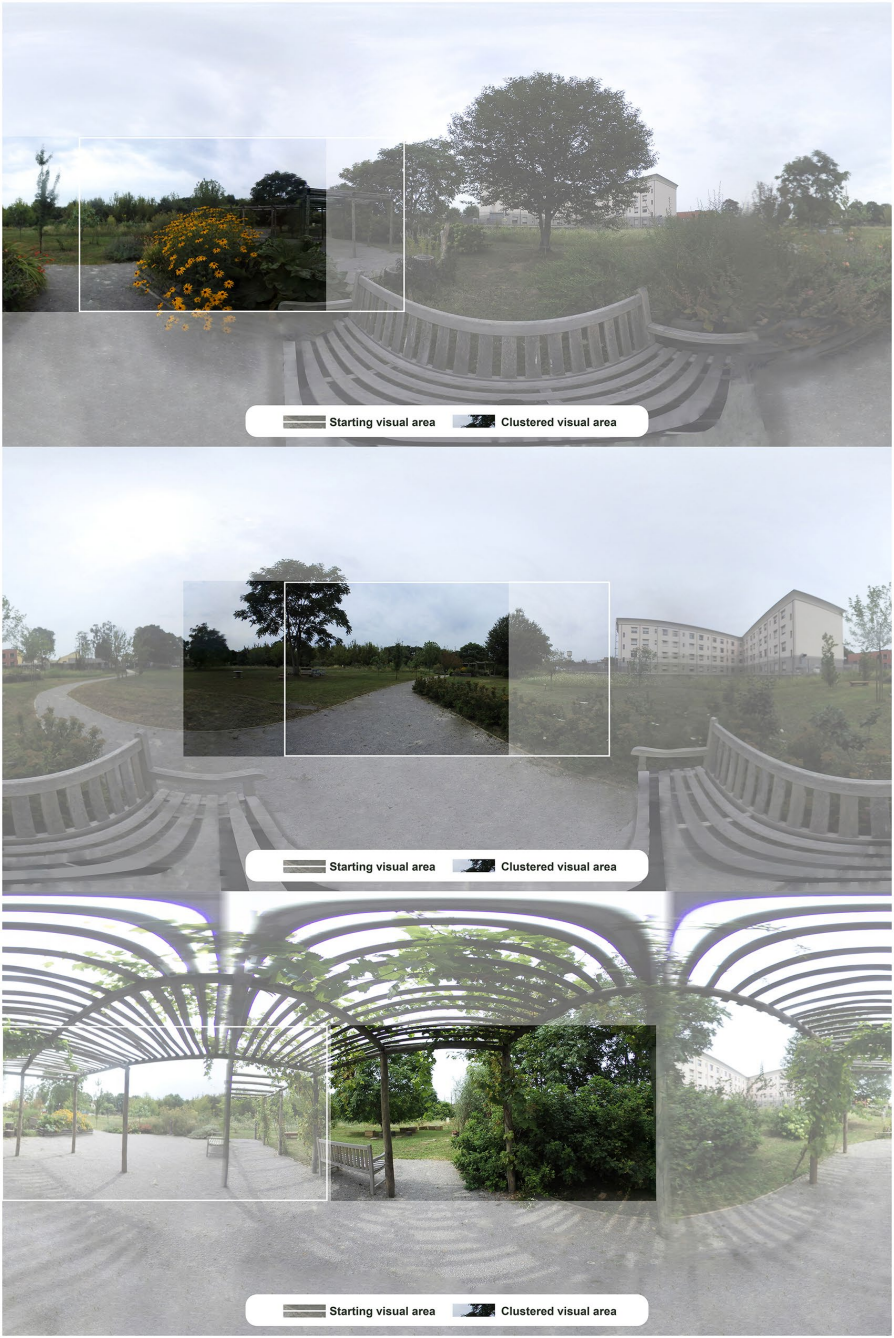
Panoramic views of POV 1 – bench on the path (top), POV 2 – bench between the flowerbeds (middle), and POV 3 – pergola (bottom). The white perimeter shows the area presented as a starting point for the visual exploration; the non-opaque area shows the target that attracted the attention of clusters 3, 4, and 1, respectively.

The 360° panoramic views virtually explored are presented unwrapped (Figure 10), to show the features of the restorative garden included in the clustered isovist previously seen on 2D maps. For each panoramic view two areas describing the visual exploration behavior are highlighted. The white-framed area indicates the initial position of the virtual camera for each POV, that is the portion of space predetermined by the authors as visible at the beginning of the digital exploration. The non-opaque is the target of the cluster corresponding to the direction that attracted the most attention and represents the average visual exploration behavior enacted by the people included in the cluster. Cluster 1 shows the largest change from the beginning to the end of the exploration, as the two areas do not overlap at all, offering completely new information about the most attractive target in contrast with the expectations of the authors. Cluster 3 and cluster 4 are instead partially consistent with the predicted behavior, given that the initial and final areas are mostly overlapping in both cases. These are the landscapes chosen by the participants for the assessment, whose results are analyzed below.

One-way repeated-measures ANOVAs are conducted to determine whether there are statistically significant differences within-groups in restoration, pleasure, and arousal over the three main clusters for each POV. Descriptive analyses are reported in Table 1. In all three clusters analyzed, positive restoration scores emerge. The scores are the highest for cluster 4.

**Table 1:** Means (M) and Standard deviations (SD) for the dependent variables over the three clusters conditions (*N* = 71).

**Variables**	**POV 1 – Cluster 3** **M (SD)**	**POV 2 – Cluster 4** **M (SD)**	**POV 3 – Cluster 1** **M (DS)**
Restoration	3.121 (0.06)	3.264 (0.06)	3.164 (0.06)
Pleasure	1.766 (0.19)	2.280 (0.18)	2.190 (0.17)
Arousal	1.170 (0.22)	1.379 (0.22)	1.218 (0.20)

Regarding restoration, the assumption of sphericity is not violated, as assessed by Mauchly’s Test of Sphericity, *p* = 0.334. The different POVs elicit statistically significant changes in restoration intensity, *F*(2, 140) = 5.430, *p* < 0.005, *η*^2^ = 0.072. *Post-hoc* analysis with a Bonferroni adjustment reveals that the level of restoration associated to cluster 4 (*M* = 3.26) is statistically significantly higher than the ones associated to cluster 3 (*M* = 3.12, *p* = 0.001) and to cluster 1 (*M* = 3.16, *p* = 0.048).

In the case of the analysis regarding pleasure, the assumption of sphericity is violated, as assessed by Mauchly’s Test of Sphericity, *p* = 0.000. Therefore, a Greenhouse–Geisser correction is applied (ε = 0.826). The different POVs elicit statistically significant changes in pleasure intensity, *F*(1,65, 115.6) = 6.40, *p* < 0.005, *η*^2^ = 0.084. *Post-hoc* analysis with a Bonferroni adjustment reveals that the level of pleasure associated to cluster 3 (*M* = 1,76) is statistically significantly lower than the pleasure associated to cluster 4 (*M* = 2.28, *p* = 0.015) and to cluster 1 (*M* = 2.19, *p* = 0.031).

Finally, a third repeated-measures within-group ANOVA is conducted to determine whether there are statistically significant differences in arousal over the three main clusters. The assumption of sphericity is not violated, as assessed by Mauchly’s Test of Sphericity, *p* = 0.639. The analysis shows that there is not a statistically significant change in arousal evaluation through the three clusters: *F*(2, 140) = 1.332, *p* = 0.267, *η*^2^ = 0.019.

In conclusion, the three clusters differ significantly with respect to how much they are associated with a restorative experience and to the degree of pleasure linked to them, whereas they do not differ with respect to emotional arousal. In particular, cluster 3 is associated with a particularly low level of pleasure and restoration, whereas cluster 4 turns out to have the highest scores in both dimensions.

A Pearson’s correlation coefficient is computed to assess the linear relationship between restoration, pleasure, and arousal evaluating clusters 3, 4, and 1. The analysis shows for each cluster a significant and positive correlation between these dimensions (see Table 2).

**Table 2:** Pearson’s Correlation Coefficient of restoration, pleasure and arousal in POV 1 – Cluster 3 (*n* = 108); POV 2 – Cluster 4 (*n* = 86); POV 3 – Cluster 1 (*n* = 138).

**POV 1 – Cluster 3**			
	**Restoration**	**Pleasure**	**Arousal**
Restoration	1	0.488^**^	0.542^**^
Pleasure	0.488^**^	1	0.510^**^
Arousal	0.542^**^	0.510^**^	1
**POV 2 – Cluster 4**			
Restoration	1	0.506^**^	0.456^**^
Pleasure	0.506^**^	1	0.500^**^
Arousal	0.456^**^	0.500^**^	1
**POV 3 – Cluster 1**			
Restoration	1	0.505^**^	0.189^*^
Pleasure	0.505^**^	1	0.286^**^
Arousal	0.189^*^	0.286^**^	1

* *p* < 0.05 and

** *p* < 0.01.

The results of the analysis of the diverse types of activities selected by survey participants help to gather additional information on the differences between the three clusters (Table 3). It is possible to observe that cluster 4 is the most associated with activities of interaction with nature (34.3% vs. 26% for cluster 3 and 22.7% for cluster 1), whereas cluster 3 is the most associated with activities of social interaction (29.3% vs. 19.5% for cluster 4 and 17.3% for cluster 1). Cluster 1 is the most associated to contemplative activities compared to the others (37% vs. 33.9% for cluster 4 and 23% for cluster 3).

**Table 3:** Prefigured activities for each cluster.

**Type of activities**	**POV 1 – Cluster 3**	**POV 2 – Cluster 4**	**POV 3 – Cluster 1**
Creative activities	0.7%	1.1%	2.7%
Contemplative activities	23.0%	33.9%	37.3%
Interaction with nature	26.0%	34.3%	22.7%
Social Interactions	29.3%	19.5%	17.3%
Fitness	16.7%	8.3%	12.0%
Break	4.3%	2.9%	8.0%

Further analyses are performed to examine more in depth the role of age in affecting the experience in the garden. For this purpose, we consider the data divided only by POV and not by cluster: the loss of information due to such aggregation is compensated for by the increased number of available cases, which is a sensitive aspect due to the reduced number of over 60 years old participants. Two sample *t*-tests are performed to compare restoration, pleasure, and arousal evaluations of each POV between over and under 60 years old. There are no significant differences between the two groups (Table 4).

**Table 4:** Restoration, pleasure, and arousal between over and under 60 years old.

	**POV 1**			**POV 2**			**POV 3**		
	UNDER 60 YO *n* = 93 M(DS)	OVER 60 YO *n* = 23 M(DS)	Two-tailed sign.	UNDER 60 YO *n* = 95 M(DS)	OVER 60 YO *n* = 26 M(DS)	Two-tailed sign.	UNDER 60 YO *n* = 106 M(DS)	OVER 60 YO *n* = 32 M(DS)	Two-tailed sign.
Restoration	3,17 (0,53)	2,93(0,75)	0.080	3,30(0,52)	3,09(0,71)	0.096	3,23(0,59)	3,13(0,63)	0.397
Pleasure	1,80 (1,6)	1,43(2,26)	0.367	2,17(1,73)	2,04(2,02)	0.755	2,01(1,54)	2,14(1,62)	0.674
Arousal	1,22 (1,75)	1,64(1.96)	0.311	1,32(1,84)	1,20(2)	0.777	1,22(1,78)	1,44(1,71)	0.544

* *p*-values refer to Student’s *t*-test.

With regard to the analysis carried out on the types of activities selected by participants under and over 60 years old, it is possible to observe that there are no significant differences with regard to POV 1 and POV 2, apart from fitness in POV 1 which is more selected by those under 60 years old. For POV 3, instead, over 60 years old selected significantly more activities related to interaction with nature than under 60 years old, while under 60 years old selected significantly more activities related to social interaction (Table 5).

**Table 5:** Type of activities in each POV for under and over 60 years old.

**Type of activities**	**POV1**			**POV2**			**POV3**		
	UNDER 60 YO	OVER 60 YO	*p*	UNDER 60 YO	OVER 60 YO	*p*	UNDER 60 YO	OVER 60 YO	*p*
Creative activities	1.3%	0.0%	0.58	2.3%	1.2%	1.00	2.4%	3.4%	0.70
Contemplative activities	23.6%	28.8%	0.34	33.7%	41.9%	0.16	37.8%	35.6%	0.71
Interaction with nature	36.7%	42.5%	0.34	35.7%	30.2%	0.34	22.9%	40.2%	0.00
Social Interactions	14.8%	16.3%	0.74	15.3%	14.0%	0.76	14.9%	6.9%	0.05
Fitness	17.7%	8.8%	0.05	8.9%	8.1%	0.82	12.5%	10.3%	0.59
Break	5.9%	3.8%	0.59	4.0%	4.7%	0.76	9.4%	3.4%	0.11

* *p*-values refer to Chi-squared test (*n* ≥ 5) and Fisher’s exact test (*n* < 5).

## Discussion and conclusion

### Information from a visual post occupancy evaluation

The visual POE presented in the current study explores the consistency between design goals and actual experience of people, mediated through virtual reality photography, in a restorative garden. The applied methodology allows us to investigate four central examination points (Sidenius et al., 2017): identification of distinctive locations and the related visual exploration, the experience of the environment described through restoration and emotions, the prefigured activities, and the health outcomes resulting from restoration and emotions. The overall experience is characterized by good levels of restoration and a positive emotional state, which are in line with the goals of the landscape designers. Focusing on the three POVs defined by the authors, the analyses offer behavioral, restorative, and emotional information about the subjective visual experience throughout the garden, which confirm the coherence between the designers’ goals translated into the physical project and the simulated visual experience of users. POV 1 is located at a wooden bench, inserted in a rest area halfway along the main path connecting the entrance of the garden to the central small square, which is designed mainly for functional purposes. This affects the experience of visitors which appears less absorbing and peculiar compared to other spots in the garden. In fact, POV 1 is associated to the highest number of clustered isovists (3, 5, and 6, even though cluster 3 includes most of the observers), which suggest an environment with a less attractive panorama and the tendency to be more distracting for the observer. This visual exploration behavior is consistent with lower values of restoration and pleasure observed for cluster 3 of POV 1, the most chosen by participants and the only one from POV 1 oriented toward the inner part of the garden. However, the overall emotional experience of cluster 3 is positively connotated, corresponding to an elated state, as observed for cluster 6; cluster 5, oriented toward the urban context, is instead characterized by a mildly sad emotion. Cluster 3 is mainly associated to social interactions, which represent the highest value among all clusters. POV 2 corresponds to the second wooden bench along the path, surrounded by two raised flowerbeds facilitating the direct contact with plants and improving the feeling of immersion: in this location, the principles of restoration have the highest influence on the design choices. As a result, the number of clustered isovists decreases and two main preferred targets emerge (clusters 2 and 4). Cluster 4, including the majority of participants from this POV and oriented toward the inner part of the garden and the small square, shows the highest values of restoration and pleasure, being the spot with the more intensely elated experience. Consistently with such psychological values, the most chosen prefigured activities are related to interaction with nature, which reach the highest value for this cluster compared to the others. POV 3 is placed under the pergola at the intersection between the small square and the playground, which is conceived as the main area for social and community activities surrounded by a rich and diverse natural landscape. Only one clustered isovist is identified for this POV, and it is oriented toward the playground rather than the center of the small square as expected by the authors. Cluster 1 shows good levels of restoration, despite the difference with cluster 4 varies according to the type of analysis. The emotional experience is very positive and consistent with that observed for cluster 4, sharing the same level of pleasure and arousal that result in an elated state. The most selected activities are contemplative, whereas social interactions do not appear strongly represented here.

In terms of behavioral reactions to the virtual simulations of the garden, it is interesting to notice that in none of the three POVs and related clusters, the initial perspective, pre-defined by the researchers as relevant views according to the features of the designed garden, was equal to the main one emerging from the cluster analysis. In all cases, the main target of the initial views was looking at the redesigned garden, thus including the path and structures, such as the pergola and the benches; instead, the main target views depicting the average reaction of people shift to frame the more natural parts of the garden, where the amount of greenery and trees is higher. This is consistent with the literature on ART, suggesting that natural elements exert a spontaneous and effortless attraction of attention in the observer (Kaplan and Kaplan, 1989). It is worth noting that the visual exploration is done on the horizontal axis, as it generally happens in reality when there are no emerging attractive points in the vertical view, such as a skyscraper or bell towers, i.e., when “the upward glance is important to give a sense of the object’s dimensions relative to the viewer” (Bosselmann, 1998, p. 171). In the study, attractive points are indeed within the garden, even if the built environment of the context, and in particular the RSA building which is looming over it, is visible in the panoramas. The exclusion of the existing urban fabric from the perspectives selected by participants seems to highlight that the landscape project manages to keep the attention and immersion of users within the green area, collaborating in reducing the impact of such buildings on the restorative effect. A deeper investigation on this hypothesis is needed: repeating the same assessment recording eye-tracking data would enable a better understanding of the impact of the existing context on the exploration task.

Eye-tracking analysis would help also in interpreting restoration results, as the higher degree of restoration in POV 2 – cluster 4 can be associated to different physical environmental features. The main difference between POV 2 and the other POVs lies in being surrounded by raised flowerbeds, which increase the feeling of immersion in nature and make the plants more accessible-also for visitors on a wheelchair-unlike all other plants in the garden. In addition to this, it is worth noting that the flowerbed included in the visual target of cluster 4 is the one with a noticeable presence of yellow flowers, which might play a role in attracting the attention and increasing perceived restoration (Todorova et al., 2004; Lindal and Hartig, 2015). The emotional reaction can be described in slightly different terms according to the collected data. Looking separately at the values of pleasure and restoration for clusters 1, 3, and 4, the only significant difference is observed for the lowest level of pleasure in POV 1 – cluster 3, whereas no differences are found for arousal. Considering instead all six clusters through descriptive statistics, five of them share a similar emotional state of elation except for cluster 5 that is mildly sad. In both descriptions we can recognize a consistent emotional experience in the garden, suggesting that the designed environment succeeds in favoring a positive emotional state. It is worth noting that, whereas the six clusters share values above the middle value on the restoration scale, cluster 5 has instead a negative result on the emotional assessment. We do not have an explanation for the effect exerted by the observed landscape, an industrial urban context, only on the emotional factors and not on the restorative ones, which might be further explored considering not only the specific target included in the isovist but also the previous short term environmental experience (Piga and Morello, 2015). This aspect requires further studies also considering the relationship between restoration and emotions observed in the current study: the positive correlation between restoration and pleasure is consistent with previous studies, whereas the positive correlation between restoration and arousal appears unexpected (Korpela and Hartig, 1996). Even though the observed levels of arousal are on average low, and the restoration and activities results appear consistent with relaxing and contemplative experience of nature; future investigation should clarify these results. From a theoretical point of view, ART (Kaplan and Kaplan, 1989) makes a distinction between soft and hard fascination: the former occurs where a sufficient level of aesthetic is present, as in natural settings, and can provide a full restorative effect. However, also hard fascination enables people to avoid using directed attention, offering a restorative effect that is more impactful for attentional recovery than for reflection on life’s larger questions (Herzog et al., 1997). Although soft fascination is assumed to be most conducive to restoration on both dimensions of attention and reflection, a form of restoration might be achieved also by more intense fascinations, particularly those that fit in or contribute to a sense of extent (Hartig et al., 1997). Since the type of fascination is connected to the quality of the environment and the duration of the exposure required, which is longer for soft fascination (Herzog et al., 1997), we can hypothesize that in our study the visual exploration through VR photography evoked a hard fascination conducive to a restorative effect with higher levels of arousal. Further experimental research on this subject can provide a better understanding of the phenomenon.

A crucial aspect of our project is the visual experience in the garden of older people, who were included in the co-design phase to ensure their needs were satisfied. The results confirm their positive visual experience both in restorative and emotional terms, and also the activities selected by this sub-sample are consistent with the design goals. Moreover, a positive result consists in the lack of significant differences between younger and older participants on all psychological variables, namely restoration, pleasure, and arousal. The same trend can be observed for the activities, showing the same distribution among the three POVs except for fitness in POV 1 (more selected by younger participants), interaction with nature (more selected by older participants), and social interactions (more selected by younger participants) in POV 3. These results suggest the achievement of a universal design approach, i.e., “the design of products and environments to be usable by all people, to the greatest extent possible, without the need for adaptation or specialized design” (Connell et al., 1997; Story, 2011). This favors the access to and usage of the garden by users of different ages, which is a valuable social factor explicitly included among the needs of those involved in the co-design project (Fumagalli et al., 2020; Boffi et al., 2021).

### Limitations and future work

Our study bears some limitations which are worth mentioning. First, the virtual experience was mono-sensory despite other senses playing an important role in affecting our perception and evaluation of the environment, especially considering that in healing gardens sound-, smell-, and thermal scapes are particularly relevant (Cooper Marcus and Sachs, 2013). Hence future studies should include more environmental features in the assessment. In addition to visual stimuli, sound appears to be the most investigated sensory channel, followed by olfactory experience (Browning et al., 2021). In our study, the comparison between the visual experience and the actual onsite exploration could be particularly affected by sound in POV 1, which is the closest to the road and the industrial area, and by smell in POV 2, which is surrounded by raised flowerbeds. Soundscape is particularly relevant for a reliable generalization of our findings to the actual restorative garden, considering that some of its parts exceed the recommended level of 55 dBA for quiet areas (Nilsson and Berglund, 2006), as a restorative garden should be. The increased effort in creating a reliable multisensory simulation must be considered, especially when one aims at applying this method to actual urban transformations rather than purely academic investigations, due to time and budget constraints. In addition, the evaluation given by participants of the panoramas is based on pictures taken on a sunny day of a specific season, i.e., spring. The seasonal dimension is a crucial element to consider in this study and when assessing green areas, as it affects the weather conditions, which exert an influence on the environmental perception (Felnhofer et al., 2015), as well as the state of plants and vegetal elements in general, influencing the restorative and preference values associated with the environment (Wang and Zhao, 2020). It is relevant to recall that in the pictures the pergola is without movable furniture (due to pandemic conditions the park was not in use at the time of shooting), and people are not present in the garden. In addition, it is relevant to notice that the experience of the study was individual: the visual exploration behavior should be further investigated when visiting the garden in couples or in groups since this can affect the way people behave in a space (Enssle and Kabisch, 2020) and what they look for in a natural public environment. All those environmental features might affect the social aspect of the assessment, for example shifting the choice of the activities from social interaction to more contemplative or nature-oriented activities, or affect the perceived restoration (Staats and Hartig, 2004). All the mentioned variables should be assessed in future studies to gain a more comprehensive understanding of the area and would be a fruitful support to local administration in managing the garden. The comparison of this study and the results of future assessments involving different variables (e.g., seasons, people, weather conditions) would be relevant for informing the method and advancing the research on the subject. We offer some final reflections to the choice of virtual reality photography, which is the basis of many limitations discussed above. In the case of preliminary phases of a design project, environmental simulations offer the advantage of sharing with citizens some design proposals in an intuitive manner, including the collection of subjective reactions. Yet, when it comes to POE, the expectation would be to collect data onsite in the physical environment. In our case, the limitations brought by the pandemic and the constraints of the project suggested to apply the exp-EIA© method, but we consider such circumstances as a particular case of a broader situation. There might be occasions when specific temporal limits for the POE contrast with a temporary inaccessibility to the area (e.g., deadlines defined by contracts between the parties involved in the environmental renovation or the institutions/community need for accessing information, temporary inaccessibility due to ineffective alignment with other landscape interventions that prevent the access to the area or difficult weather conditions), hence having the chance of anticipating a visual POE through virtual reality would offer the advantage of having preliminary data, before developing a traditional POE onsite. In such perspective, the proposed exp-EIA© method can be fruitfully applied also in the actual physical environment, as it is designed for integration into an app for mobile devices (Piga et al., 2021b). This would allow a direct comparison of data, overcoming the limitation of the pre-determined POVs applied in this study and letting the participants free to explore the environment.

## Data availability statement

The raw data supporting the conclusions of this article will be made available by the authors, without undue reservation.

## Ethics statement

The studies involving human participants were reviewed and approved by the University of Milan. The patients/participants provided their written informed consent to participate in this study.

## Author contributions

NF, GSe, PI, and MB: conceptualization. NF, GSe, PI, MB, and BP: methodology. GSt: software. NF and EF: landscape design. BP: validation. MB, LP, and GSt: formal analysis. MB, BP, GSt, and LP: investigation. LP and GSt: data curation. MB, EF, and LP: writing—original draft preparation. NF, MB, and BP: writing—review and editing. BP, GSt, and EF: visualization. PI and GSe: supervision. NF: funding acquisition. All authors contributed to the article and approved the submitted version.

## Funding

This work was supported by Fondazione Cariplo (Aging and social research: people, places and relation, 2018).

## Conflict of interest

The authors declare that the research was conducted in the absence of any commercial or financial relationships that could be construed as a potential conflict of interest.

## Publisher’s note

All claims expressed in this article are solely those of the authors and do not necessarily represent those of their affiliated organizations, or those of the publisher, the editors and the reviewers. Any product that may be evaluated in this article, or claim that may be made by its manufacturer, is not guaranteed or endorsed by the publisher.

## Footnotes

^1^ www.marzipano.net
